# Changes in Membrane Ceramide Pools in Rat Soleus Muscle in Response to Short-Term Disuse

**DOI:** 10.3390/ijms20194860

**Published:** 2019-09-30

**Authors:** Alexey M. Petrov, Maria N. Shalagina, Vladimir A. Protopopov, Valeriy G. Sergeev, Sergey V. Ovechkin, Natalia G. Ovchinina, Alexey V. Sekunov, Andrey L. Zefirov, Guzalia F. Zakirjanova, Irina G. Bryndina

**Affiliations:** 1 Institute of Neuroscience, Kazan State Medical University, Butlerova st. 49, 420012 Kazan, Russia; zefiroval@rambler.ru (A.L.Z.); gffysiology@gmail.com (G.F.Z.); 2 Laboratory of Biophysics of Synaptic Processes, Kazan Institute of Biochemistry and Biophysics, Federal Research Center “Kazan Scientific Center of RAS”, P. O. Box 30, Lobachevsky Str., 2/31, 420111 Kazan, Russia; 3 Department of Pathophysiology and Immunology, Izhevsk State Medical Academy, Kommunarov St. 281, 426034 Izhevsk, Russia; uvula@mail.ru (M.N.S.); vladimirvst@yandex.ru (V.A.P.); ovechkin-sv@mail.ru (S.V.O.); ntly.82@mail.ru (N.G.O.); d1key@inbox.ru (A.V.S.); i_bryndina@mail.ru (I.G.B.); 4 Department of Physiology, Cell Biology and Biotechnology, Institute of Natural Science, Udmurt State University, University St. 1, 426034 Izhevsk, Russia; cellbio@yandex.ru

**Keywords:** skeletal muscle, disuse, ceramide, sphingomyelinase, TNFR1, lipid rafts, neuromuscular junction, membrane lipid asymmetry

## Abstract

Lipid raft disruption is an early event during skeletal muscle unloading. Ceramide (Cer) serves as a signaling lipid that can contribute to lipid raft disturbance and muscle atrophy. Using biochemical and fluorescent approaches, the distribution of Cer and related molecules in the rat soleus muscle subjected to 12 h of hindlimb suspension (HS) was studied. HS led to upregulation of TNFα receptor 1 (TNFR1), Cer-producing enzymes, and acid and neutral sphingomyelinase (SMase) in detergent-resistant membranes (lipid rafts), which was accompanied by an increase in Cer and a decrease in sphingomyelin in this membrane fraction. Fluorescent labeling indicated increased Cer in the sarcoplasm as well as the junctional (synaptic) and extrajunctional compartments of the suspended muscles. Also, a loss of membrane asymmetry (a hallmark of membrane disturbance) was induced by HS. Pretreatment with clomipramine, a functional inhibitor of acid SMase, counteracted HS-mediated changes in the Cer/sphingomyelin ratio and acid SMase abundance as well as suppressed Cer accumulation in the intracellular membranes of junctional and extrajunctional regions. However, the elevation of plasma membrane Cer and disturbance of the membrane asymmetry were suppressed only in the junctional compartment. We suggest that acute HS leads to TNFR1 and SMase upregulation in the lipid raft fraction and deposition of Cer throughout the sarcolemma and intracellularly. Clomipramine-mediated downregulation of acid SMase can suppress Cer accumulation in all compartments, excluding the extrajunctional plasma membrane.

## 1. Introduction

Prolonged space flight, bed rest, and immobilization inevitably lead to varying degrees of muscle wasting despite preventive countermeasures [1,2,3,4,5,6]. To simulate muscle unloading, the most commonly used method is hindlimb suspension (HS). This is a generally accepted model for the development of disuse muscle atrophy and dysfunction [7,8,9,10].

Numerous studies have elucidated the morphological, functional, and biochemical changes that are inherent in atrophied muscles and develop after 4–7 days of disuse. However, mechanisms of the observed atrophic process in the disused skeletal muscles would be better estimated and comprehended when being studied in both initial and advanced stages of the unloading. Although the alterations of some parameters may differ substantially in the beginning of muscle unloading in comparison with long-term exposure, early events may become the key points in triggering muscle atrophy [11,12,13,14,15,16,17,18,19,20]. 

Pharmacological intervention in sphingolipid metabolism is a promising therapeutic strategy for the treatment of neuromuscular disorders. To date, there is increasing evidence pointing to the important role of sphingolipids, including their backbone molecule ceramide (Cer), in the regulation of skeletal muscle function [21,22]. Cer accumulation in skeletal muscles has been detected in response to numerous nutritional and stressful stimuli, such as a high-fat diet, free fatty acid oversupply, fasting, reperfusion, and oxidative damage [23,24,25]. We have previously demonstrated [26] that the amount of Cer increases in rodent soleus muscle subjected to acute and long-term HS. Some studies conducted both on rats and humans have also confirmed muscular Cer accumulation during disuse [27,28,29].

It is known that Cer is produced in cells through three main pathways: de novo synthesis, hydrolysis of sphingomyelin by sphingomyelinases (SMases), and sphingosine reacylation (salvage pathway) [30]. Our data indicated that in a disused soleus muscle, Cer can be generated preferentially by SMase-mediated hydrolysis [31]. SMases may act in different cellular compartments [32] and their effects on membrane order have been described in model and cellular membranes [33,34]. Produced Cer can form Cer-enriched microdomains which then merge spontaneously into large platforms, thereby facilitating the clustering of signaling molecules translating stress-related signals [35]. Acid SMase (aSMase) may act synergistically with neutral SMase (nSMase) [36]. Both aSMase and nSMase activity may be induced by TNFα [37,38], and this effect of TNFα has been shown in skeletal muscles [39,40].

Sphingomyelin hydrolysis by the SMases considerably affects cholesterol- and sphingolipid-rich membrane microdomain (raft) integrity, and lipid raft cholesterol displacement by Cer could be one of the mechanisms [41,42]. Another pathway of Cer action in plasma membrane is a loss of normally existing lipid asymmetry, a crucial factor required for maintenance of mechanical stability of the membrane, myotubule formation, vesicular transport, and signal transduction [43,44,45,46].

Previously, we demonstrated substantial lipid raft disassembly in rat soleus muscles subjected to 6–12 h HS [17]. Importantly, pretreatment with clomipramine, belonging to the family of functional inhibitors of acid sphingomyelinase (FIASMA), selectively promoted the retention of raft integrity in the synaptic (junctional) regions [18]. We hypothesized that the initial period of HS is accompanied by Cer accumulation within lipid rafts due to SMase activation; furthermore, the junctional and extrajunctional compartments could have specific features of Cer deposition due to unique functional and metabolic properties. To test this hypothesis, using biochemical methods and fluorescent labelings, we studied the Cer distribution in the junctional and extrajunctional regions as well as the changes in Cer, sphingomyelin, aSMase, nSMase, and TNFα receptor 1 (TNFR1) content in the lipid raft fraction of suspended soleus muscles. In parallel, the efficacy of clomipramine pretreatment and alterations in the lipid asymmetry were tested.

## 2. Results

### 2.1. Immunofluorescent Detection of Ceramide Distribution on Transverse Sections

**Figure 1:**
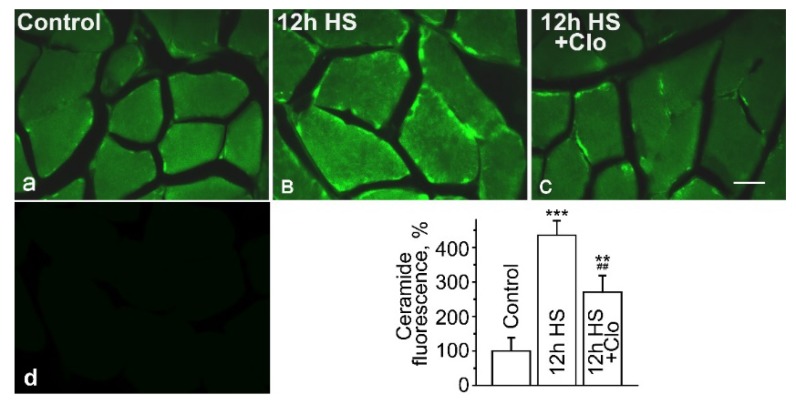
Expression of immunoreactive ceramide (Cer) on transverse sections of soleus muscle fibers in control rats (**A**), rats after 12 h of hindlimb suspension (HS) (**B**) and 12 h of HS with clomipramine (Clo) pretreatment (**C**). Scale bar—100 µm. Image (**D**) represents the negative control. The graph is the quantification of ceramide fluorescence (mean ± SEM). Data are shown as a percentage of the baseline value (100%) obtained in the control group. ** *p* < 0.01 and *** *p* < 0.001 denote statistically significant differences in comparison with the control value; ^##^ *p* < 0.01—the difference between non-pretreated and clomipramine-pretreated groups. Control muscles—Control; muscles from HS for 12 h rats—HS; muscles from HS and clomipramine-pretreated animals—HS + Clo. *n* = 5–6 animals for each group.

The results of the immunohistochemical study are presented in Figure 1. On transverse sections of the muscle fibers of control rats (*n* = 5), the immunoreactive labeling of Cer had the form of a diffuse signal in the perinuclear areas of the sarcoplasm and a network of rare and slightly colored point clusters of small size in the vicinity of the plasma membrane (Figure 1A). HS led to a significant increase in ceramide immunoreactivity (Figure 1B). The total fluorescence intensity, measured at standard areas (0.8 mm^2^), was enhanced in the suspended muscles (*n* = 5), making up 436% ± 43% of the control (*p* < 0.001). The most intense labeling was located in the perinuclear region. Increased fluorescence was determined both in clusters near the sarcolemma and in the sarcoplasm, where immunoreactive Cer looked like a thin network over the entire diameter of the fibers. In the muscles of clomipramine-treated rats (*n* = 6) exposed to HS, the total labeling intensity was lower than in nontreated animals (*p* < 0.01) but still significantly exceeded the control level (*p* < 0.01). Localization of the immunoreactive Cer in muscles of the clomipramine-administered HS group was similar to that in freely moving animals (Figure 1C). Note that if anti-Cer antibodies were not used in the staining protocol, negligible immunofluorescence was detected (Figure 1D). 

The obtained results indicate the accumulation of Cer in unloaded for 12 h soleus muscles. Localization of the immunolabeling suggests that Cer storage in HS can occur both in sarcolemmal and intracellular compartments of muscle fibers. The increase of plasma membrane Cer is consistent with our previous data, which demonstrated the formation of micrometer-scale membrane domains after 4 days of muscle disuse [47].

### 2.2. Biochemical Analysis of Isolated Lipid Raft Composition 

**Figure 2:**
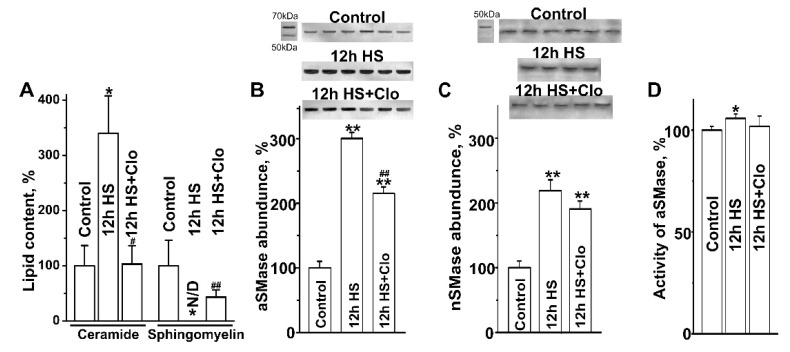
Hindlimb suspension increased the amount of Cer and SMases in detergent-resistant membrane (DRM) fraction of soleus muscle: effect of clomipramine pretreatment. (**A**) Cer and sphingomyelin levels. (**B**) and (**C**) aSMase and nSMase content in DRM fractionassessed by Western blot. (**D**) aSMase activity in muscle homogenates. Data (mean ± SEM) are shown as a percentage of the baseline value (100%) obtained in the control group of rats. * *p* < 0.05, ** *p* < 0.01 denote statistically significant differences in comparison with the control value; ^#^ *p* < 0.05, ^##^ *p* < 0.01 denote the difference between non-pretreated and clomipramine-pretreated groups. Control muscles—Control; muscles from HS rats—HS; muscles from HS and clomipramine-pretreated animals—HS + Clo. *n* = 4–6 animals for each group.

Previously, using a biochemical approach, we revealed that Cer accumulates in rodent soleus muscle after 12 h, 4 days, 14 days, and 30 days of HS, and the main mechanism may be associated with SMase-dependent hydrolysis of sphingomyelin [26,31]. It has been shown that Cer can displace cholesterol from membrane of lipid rafts, leading to their disassembly [41,48]. Given that lipid raft disruption is an early event during muscle disuse [17] and functional aSMase inhibitor clomipramine partially prevents this effect [18], we suggest that lipid rafts might be one of the main sites of Cer production in response to HS. To test this possibility, the detergent-resistant membrane fraction containing lipid rafts was isolated for biochemical study. Here, we found that 12 h of HS led to significant changes in Cer, sphingomyelin, aSMase, and nSMase levels in the raft-containing fraction of the suspended muscles. First, HS was accompanied by an increase in Cer availability (by 240% ± 68%, *p* < 0.05, *n* = 5, versus control, *n* = 6; Figure 2A). In contrast, sphingomyelin levels dramatically declined after HS below the detection limit (*p* < 0.01, *n* = 5, versus control, *n* = 6). This suggests a strong reversal correlation between raft Cer and sphingomyelin in the suspended muscle. Second, in the detergent-resistant membrane fraction, the abundance of aSMase and nSMase increased (by 200% ± 10%, *p* < 0.01, *n* = 6, and by 118% ± 15%, *p* < 0.01, *n* = 5, respectively) in suspended muscles compared with the control (Figure 2B,C). Thus, aSMase and nSMase were significantly upregulated and both of them may be responsible for HS-induced Cer overproduction within the lipid rafts. In addition, aSMase activity measured in total muscle homogenate (*n* = 6 for each group) was slightly but statistically significantly increased after HS (Figure 2D).

Clomipramine pretreatment (1) abolished Cer accumulation in the lipid raft fraction, whereas the drop in the sphingomyelin level was only partially prevented (Figure 2A); (2) attenuated the effects of unloading on aSMase content (Figure 2B), which decreased by 28% ± 3% (*p* < 0.01, *n* = 6) compared with HS; and (3) restored aSMase activity (Figure 2D). Note that clomipramine did not significantly affect nSMase availability (Figure 2C).

**Figure 3:**
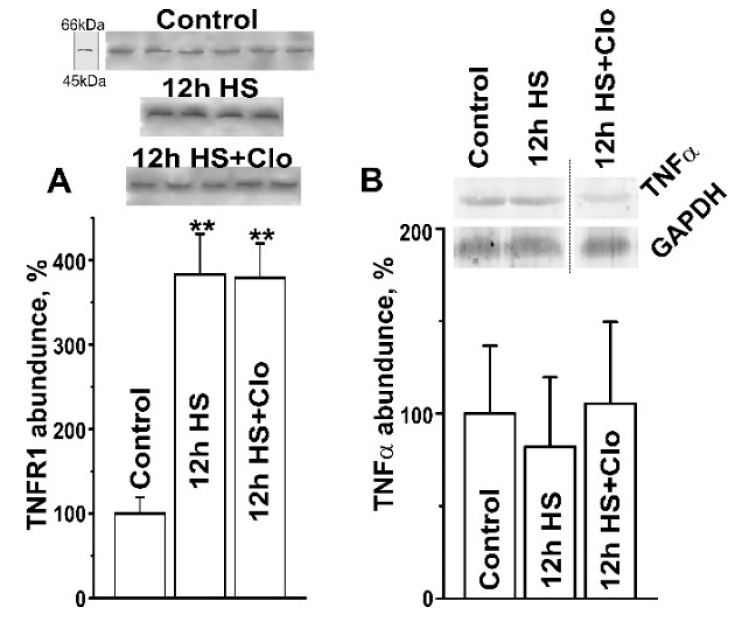
Hindlimb suspension increased the TNFα receptor 1 (TNFR1) content in DRM fraction but did not change the TNFα content in soleus muscle homogenates. (**A**) and (**B**) TNFR1 and TNFα content. (**B**) Quantification of the relative TNFα expression after normalization to the GAPDH signal is shown. Data (mean ± SEM) are presented as a percentage of the baseline value (100%) obtained in the control group of rats. ** *p* < 0.01 denotes statistically significant differences in comparison with the control value. *n* = 4–6 animals for each group. Other details are as in Figure 2.

In skeletal muscles, expression of aSMase and nSMase may be upregulated in response to activation of TNFR1 by TNFα [39,40]. Indeed, the TNFR1 content in the detergent-insoluble fraction of the plasma membrane increased almost 4-fold (*p* < 0.01) after HS, but clomipramine treatment did not affect it (Figure 3A). This suggests that increased expression of TNFR1 could trigger SMase upregulation, and clomipramine can probably act downstream of TNFR1. Interestingly, the TNFα content in muscle homogenates did not change in HS or HS with clomipramine pretreatment (Figure 3B).

### 2.3. Fluorescent Analysis of Cer Distribution with Confocal Microscopy

**Figure 4:**
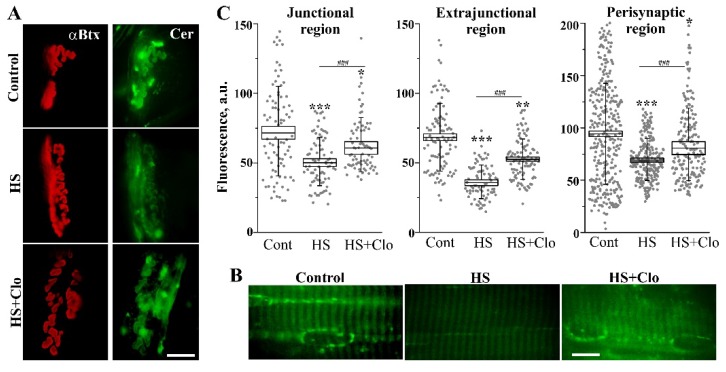
HS decreased the membrane staining with fluorescent BODIPY FL C5-Ceramide (BODIPY-Cer): influence of clomipramine pretreatment. (**A**) Junctional regions double-labeled with α-bungarotoxin (α-Btx) and BODIPY-Cer in control, suspended muscle (HS), or suspended muscle of clomipramine-pretreated rats (HS + Clo). Additionally, the green fluorescent spots are visualized in the region surrounding the synaptic zone (perisynaptic region). (**B**) BODIPY-Cer fluorescence in the extrajunctional regions of the muscle fibers. (**A**) and (**B**) scale bars—10 μm. (**C**) The box plots indicate the changes in the fluorescent BODIPY-Cer signal in the junctional, extrajunctional, and perisynaptic regions in control, suspended nontreated, and clomipramine-treated muscles. Gray spots represent individual measurements (12–53 measurements per animal and *n* = 6 different animals per group). The measurements were pooled together to obtain the mean values (the central horizontal lines of the boxes). Standard errors (box ranges) and standard deviations (whiskers) are shown. *Y*-axis—intensity of green fluorescence in a.u. * *p* < 0.05, ** *p* < 0.01, *** *p* < 0.001 are statistically significant differences compared with the corresponding control value. ^###^ *p* < 0.001 is between nontreated and clomipramine-treated groups.

**Figure 5:**
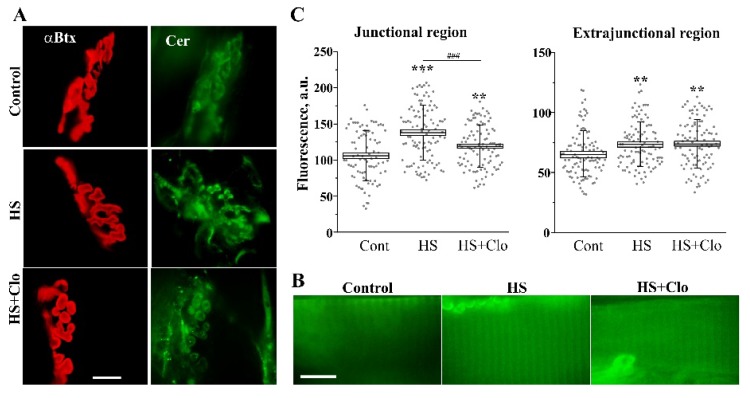
HS increased the immunofluorescent labeling of plasma membrane ceramide: junctional-specific effect of clomipramine therapy. (**A**) and (**B**) The fluorescent images of junctional (**A**) and extrajunctional (**B**) regions. Membrane Cer was labeled with anti-Cer antibody (green channel) in control, suspended muscle (HS), or suspended muscle of clomipramine-treated rats (HS + Clo). α-Btx (red channel) was used for localization of nicotinic acetylcholine receptors (nAchRs) in postsynaptic membranes. Scale bars—10 μm. (**C**) The box plots show the alteration of ceramide immunofluorescent staining (in a.u.) in junctional/extrajunctional compartments in the control, suspended nontreated, and clomipramine-treated muscles. Gray spots represent individual measurements (14–20 measurements per animal and *n* = 6 different animals per group). ** *p* < 0.01, *** *p* < 0.001 are statistically significant differences compared with the corresponding control value. ^###^ *p* < 0.001 is between nontreated and clomipramine-treated groups. Other details are as in Figure 4.

Junctional and extrajunctional membranes have different biochemical and functional properties, including lipid raft composition and cholesterol content [48,49,50,51,52]. So, it is important to study the distribution of Cer in the junctional and extrajunctional compartments. To detect Cer in different regions of muscle fiber membranes, we used two fluorescent approaches based on the application of exogenous BODIPY FL C5-Ceramide (BODIPY-Cer) or immunofluorescent staining of endogenous Cer in the plasma membranes. In both cases, the junctional (synaptic) area was stained with fluorescent α-bungarotoxin (α-Btx) for specific labeling of the postsynaptic receptors (Figure 4 and Figure 5).

BODIPY-Cer is mainly concentrated in the endoplasmic reticulum (ER) and Golgi complex of living cells [53,54,55]. The incorporation of BODIPY-Cer to synaptic membranes depends on the level of endogenous Cer, and uptake of fluorescent Cer is greater by ceramide-poor membranes [49,55]. HS markedly decreased the membrane staining with the exogenous Cer (Figure 4) in both the junctional and extrajunctional regions (*n* = 6 animals versus *n* = 6 in the control). Additionally, fluorescence in the perisynaptic region was detected, where the fluorescence was concentrated in spots. The fluorescence of spots was higher in the control muscles (Figure 4A). These spots most likely represent an accumulation of the labeled Cer in the ER of perisynaptic Schwann cells. Clomipramine pretreatment was able to partially prevent changes in BODIPY-Cer fluorescence in the junctional, extrajunctional, and perisynaptic compartments of the suspended muscles (*n* = 6 animals). The decrease in BODIPY-Cer fluorescence after HS and prevention of this by clomipramine suggest that HS is associated with the accumulation of endogenous Cer in the junctional/extrajunctional/perisynaptic membranes, including the ER, and activity of aSMase might be required for the increase in the endogenous Cer content. Note that clomipramine treatment itself did not modify total cholesterol and Cer levels and lipid raft integrity in the junctional and extrajunctional membranes [18].

Immunofluorescent labeling of plasma membrane Cer (without permeabilization of the cell membrane with a detergent) revealed increased fluorescence in junctional and extrajunctional membranes after HS (*n* = 6 animals versus *n* = 6 animals in the control; Figure 5). Interestingly, near and inside the synaptic region, fluorescent spots were visualized (Figure S1). These spots might reflect the formation of Cer platforms, some of which could be released in the form of extracellular vesicles [56]. Clomipramine pretreatment partially decreased this enhancement of Cer fluorescence and spot formation in the junctional but not extrajunctional region (*n* = 6 animals). Interestingly, the immunofluorescent staining of extrajunctional membranes was similar after HS alone and in combination with clomipramine pretreatment (Figure 5B,C). Additionally, HS markedly increased the fluorescent signal from the perisynaptic regions, but it was not markedly reversed by clomipramine treatment (Figure 5A).

These data suggest that HS can increase Cer levels throughout the plasma membrane and large clusters of Cer appear within the synaptic region, containing pre- and postsynaptic membranes as well as perisynaptic glial cells. Probably, the accumulation of Cer in the synaptic plasma membranes tightly depends on aSMase activation. Note that if muscles were incubated only with the secondary antibody, no specific fluorescent staining was observed (Figure S2).

### 2.4. Estimation of Membrane Asymmetry Using F2N12S Probe

**Figure 6:**
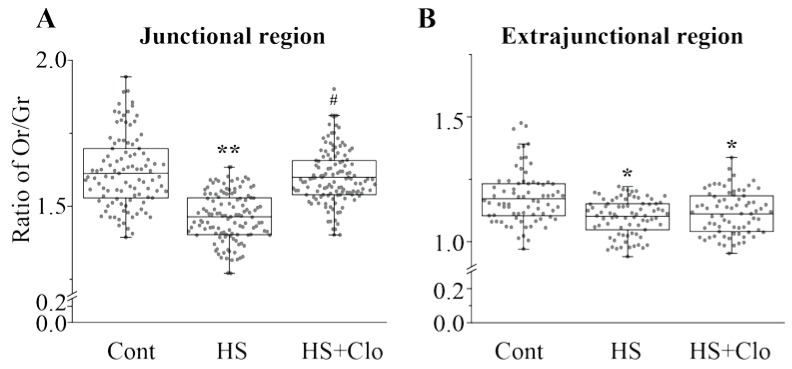
HS affected the ratio of orange (Or) and green (Gr) fluorescence of F2N12S: effect of clomipramine treatment. Or and Gr fluorescence of F2N12S was measured in junctional (Btx-positive) (**A**) and extrajunctional (**B**) regions, and the ratio of Or/Gr signals was then calculated in control (Cont), suspended (HS) muscle, and suspended muscle of rats treated with clomipramine (HS + Clo). A decrease in Or/Cr is an indicator of loss of plasma membrane asymmetry. (**A**) and (**B**) show the box plots illustrating changes in the Or/Gr ratio. Gray spots represent individual measurements (12–22 measurements per animal and *n* = 6 different animals per group). Other details are as in Figure 3. * *p* < 0.05, ** *p* < 0.01 are statistically significant differences compared with the corresponding control value. ^#^ *p* < 0.05 is between nontreated and clomipramine-treated groups.

Cer accumulation could disturb membrane phospholipid asymmetry (a hallmark of membrane disturbance). Loss of membrane asymmetry may contribute to structural change in the membrane, in particular, integrity and permeability [43]. Potentially, the altered lipid asymmetry could affect neuromuscular function due to the importance of anionic lipid distribution for synaptic vesicle exo-endocytosis [57]. HS decreased the ratio of orange/green (Or/Gr) fluorescence of the F2N12S probe (see Methods for details) in both junctional (more pronounced) and extrajunctional regions, indicating disturbance of the lipid asymmetry throughout the plasma membranes (Figure 6A,B). Clomipramine treatment completely prevented the reduction of the Or/Gr ratio in the junctional membranes (Figure 6A), whereas the ratio in extrajunctional membranes was not altered significantly (Figure 6B). This suggests a synapse-specific action of clomipramine. Note, that the Or/Gr ratio was lower in the extrajunctional membranes than in the junctional, demonstrating the unique features of the synaptic membranes.

## 3. Discussion

Our results suggest that acute HS leads to accumulation of Cer and a decrease in sphingomyelin in the lipid raft fraction as well as disturbance of the lipid asymmetry. This is accompanied by an upregulation of both aSMase and nSMase content in the lipid rafts, with a simultaneous increase in TNFR1. Taking into account the substantial reciprocal changes between Cer and sphingomyelin as well as the enhanced aSMase activity in muscle homogenates, we suggest that an increase in the membrane Cer pool may be associated with enhancement of sphingomyelin hydrolysis by SMases. Importantly, all these effects (excluding the increase in TNFR1 and nSMase amount) were sensitive to pretreatment with clomipramine.

Clomipramine, like some other compounds from the FIASMA family, is able to induce proteolytic degradation of aSMase, interrupting its binding to lipid bilayers within lysosomes and thereby making the enzyme vulnerable to cleavage by lysosomal proteases [58,59,60,61]. Hence, aSMase-activating stimuli can no longer trigger a translocation of the enzyme to the plasma membrane, and the downstream signaling cascade is lost upon FIASMA treatment [58,59,60,61]. Importantly, the drugs belonging to this family are widely used in clinical practice. 

There are controversial data regarding the content of TNFα in disused skeletal muscles [62,63,64]. Hunter et al. [62] did not find any changes in TNFα levels in rat soleus muscle after 7 days of HS. Hirose et al. [63] established a significant increase in TNFα in rat soleus muscle by 3, 7, and 14 days of HS compared with control groups and 1 day HS. Al-Nassan et al. [64] described the TNFα downregulating effect of exercise in murine gastrocnemius muscle disused for 6 weeks. Our experiments did not demonstrate any changes in TNFα in muscle homogenates but revealed an upregulation of TNFR1 in the lipid raft fraction of disused soleus muscle. This can reflect that increased TNFR1 levels in unloaded muscle precedes TNFα accumulation at the later stage of HS [63]. It is known that TNFα acting via TNFR1 can lead to SMase activation in different cell types, including muscle fibers [39,40]. Thus, TNFR1 overexpression in unloaded muscle may be regarded as a possible mechanism for SMase activation and one of the early events in muscle adaptation to disuse. Clomipramine did not prevent the increase in TNFR1, suggesting the downstream action of the drug. It is important to note that increased TNFR1 expression in muscle can be associated with caspase-dependent proteolysis [65].

Fluorescent labeling provides insights into the distribution of Cer in the specific muscle compartments as well as junctional and extrajunctional regions. These experiments suggest that HS can increase Cer abundance both in the junctional and extrajunctional plasmalemma as well as Schwann cell membranes. Also, the intracellular content of Cer (e.g., in ER and lysosomes) was upregulated in these regions after HS. Interestingly, Cer-positive puncta and vesicle-like structures were visualized in the junctional region of suspended muscles (Figure S1), which can reflect the formation of Cer-rich extracellular vesicles or exosome release in response to HS. Pretreatment with clomipramine (a functional inhibitor of aSMase) counteracted the rise of Cer levels selectively in the junctional (but not extrajunctional) plasmalemma and formation of Cer-positive vesicles/spots near the neuromuscular junctions. Simultaneously, the increased Cer deposition in the intracellular membranes was suppressed by the functional aSMase inhibitor. 

Probably, activation of aSMase during muscle disuse can lead to enhanced production of Cer, which accumulates in the membranes of both junctional and extrajunctional compartments. Primary sites of aSMase-driven Cer generation may be located not only in the surface membrane but also in the intracellular membrane compartments, for example, late endosome/lysosomes [66]. This is consistent with the inhibitory action of FIASMA on aSMases located in the lysosomes [59]. This can explain why the functional aSMase inhibitor effectively prevented the HS-mediated decrease in BODIPY-Cer staining in all compartments, while the enhancement of the plasma membrane Cer labeling was attenuated only at the junctional regions. High sensitivity of the synaptic compartment to inhibition of aSMase in the suspended muscle may be caused by a higher rate of Cer removal, for example, due to activity of synaptic-specific ceramidase [49] and/or high expression of acid ceramidase in this compartment [67]. On the other hand, the higher “resistance” of Cer content in the extrajunctional sarcolemma to clomipramine pretreatment may be linked with relatively high expression of nSMase in the muscle fibers, where the expression of the enzyme is decreased by muscle activity [68,69]. Simultaneously activated Cer biosynthesis in the ER or salvage pathway might be another source of Cer accumulation, which cannot be excluded. Probably, incomplete matching between biochemical and fluorescent data regarding the efficacy of clomipramine treatment could be caused by differences in local and global changes in Cer levels.

It is known that loss of membrane asymmetry is an early hallmark of cell damage [43,70] affecting neuromuscular transmission [57]. HS induced disturbance of lipid asymmetry both in the junctional and extrajunctional regions, but clomipramine pretreatment abolished this effect of HS specifically in the junctional compartment. This indicates a target action of clomipramine on the synaptic membranes.

In summary, the presented results suggest that HS can induce Cer accumulation in junctional and extrajunctional compartments. This could be mediated by an increase in SMase content, especially in lipid rafts which are disintegrated by acute disuse, as previously found [17,18]. Clomipramine accelerating aSMase degradation may counteract the upregulation of the raft Cer, most profoundly, in the junctional region. Probably, in the extrajunctional compartment, clomipramine can mainly affect intracellular Cer deposition. This is consistent with the ability of clomipramine to selectively hinder lipid raft disassembly [18] and disturbance of the lipid asymmetry in the junctional compartment. The region-specific action of the aSMase inhibitor raises the question about the unique features of Cer metabolism within the synaptic compartment.

## 4. Materials and Methods 

### 4.1. Animals

All experiments were performed on male Wistar rats (180–230 g). The study conformed to the Guide for the Care and Use of Laboratory Animals (NIH Publication No. 85–23, revised 1996) and the European Convention for the Protection of Vertebrate Animals Used for Experimental and Other Scientific Purposes (Council of Europe No. 123, Strasbourg, 1985). The experimental protocol met the requirements of the EU Directive 2010/63/EU and was approved by the Bioethics Committees of Izhevsk State Medical Academy (Protocol #3/ 29 Jan 2016). As female and male rats have different degrees of decrement in neuromuscular function [71], only males were used to decrease data variability.

### 4.2. Experimental Protocol

The experiment was performed as described previously [18]. All rats were divided into three groups. The animals of the first and second groups were subjected to HS individually in custom cages for 12 h with free access to food and water and the ability to move around the cage without support of the hind limb (tail-suspension model) [7,9]. Rats in the first group were pretreated with clomipramine (Novartis Pharma AG, Basel, Switzerland) for 5 days before HS (intramuscularly, 1.25 mg/g body weight daily); rats in the second group, instead of clomipramine, were administered with the equivalent volume of 0.9% saline solution. Control rats received no unloading and no clomipramine (third group). Previously, we used a control that was not unloaded but received clomipramine, which showed that clomipramine alone did not affect ceramide and cholesterol levels in muscle homogenates as well as lipid raft integrity [18]. Therefore, in the present work, we did not perform experiments on clomipramine-pretreated but non-unloaded animals.

Immediately after the end of the HS, the rats were anesthetized by an intraperitoneal injection of sodium pentobarbital (80 mg/kg) before decapitation with a guillotine. Soleus muscles were harvested, quickly frozen in liquid nitrogen, and stored at −80 °C before further biochemical assay. For the ex vivo fluorescent study, soleus muscles with nerve stump were placed after removal in a chamber with a physiological solution containing (mM) NaCl, 137; KCl, 5; CaCl_2_, 2; MgCl_2_, 2; NaHCO_3_, 24; NaH_2_PO_4_, 1; glucose, 11; pH 7.4. The solution was continuously bubbled with 95% O_2_ and 5% CO_2_ and maintained at room temperature. All reagents were from Sigma (Deisenhofen, Germany). 

### 4.3. Ceramide, Sphingomyelin, Sphingomyelinase, TNFR1, and TNFα Assays

Cer and sphingomyelin were assessed by high-performance thin-layer chromatography (HPTLC) in a detergent-insoluble membrane fraction isolated from the homogenates of m. soleus. To isolate the lipid-raft-containing fraction, we used a method described previously [72]. For this, the samples of muscle tissue (10 mg) were homogenized in 1 mL of lysing TBS buffer (1% Triton X-100 in 25 mM Tris/HCl + 140 mM NaCl + protease/phosphatase inhibitors, pH 7.5) with IKA T 10 Basic Ultra Turrax Homogenizer (IKA, Staufen, Germany). The homogenates were incubated at +4 °C for 30 min. The obtained lysates were mixed with 2 mL of 60% sucrose in TBS (25 mM Tris/HCl + 140 mM NaCl, pH 7.5), then sucrose (TBS) was successively layered in the order of 1 mL of 30% solution and 1 mL of 5% solution. The samples were centrifuged at 300,000× *g* for 3 h at +4 °C, and then 0.6–1.0 mL of the top fraction was collected and analyzed. Ganglioside GM1 in the isolated raft-containing fraction was used as a raft marker. For GM1 detection, chloroform extracts prepared from the raft-containing fraction were developed on HPTLC Silica gel 60 F_254_ plates (Merck, Darmstadt, Germany) with GM1 standard (Avanti polar lipids, Alabaster, AL, USA) in a propanol:water (7:3) solvent system as described earlier [73]. 

For Cer and sphingomyelin detection, lipids from the raft fraction were extracted with Folch reagent (chlorophorm:methanol, 2:1) [74] and processed as described previously [73]. Chloroform extracts (0.1 mL) were spotted on HPTLC Silica gel 60 F_254_ plates and developed in a butanol:acetic-acid:water (3:1:1) solvent system [75] together with equivalent volume of the standard chloroform solution of Cer and sphingomyelin (Avanti polar lipids, USA). Plates were imaged by iodine vapor and analyzed by a video densitometer (Sorbfil, Krasnodar, Russia) at UV light (254 nm). Cer and sphingomyelin quantitative analysis was performed with Sorbfil TLC Videodensitometer software (Sorbpolymer, Krasnodar, Russia). The values of standard samples of Cer and sphingomyelin were used for calculation. 

To assay the amount of aSMase, nSMase, and TNFR1 in the isolated lipid raft fraction as well as TNFα in muscle homogenates, Western blot analysis was used. Briefly, the raft fraction from the sucrose gradient (25 μL, concentration of total protein 10–15 mg/mL) was analyzed by SDS/PAGE as described by [76] on a 10% (*w/v*) acrylamide gel (Mini-Protean^®^, Bio-Rad, Hercules, CA, USA). Proteins from the gel were then transferred onto a nitrocellulose membrane for immunoblot analysis (Mini Trans-Blot^®^, Bio-Rad). Membranes were incubated overnight with diluted primary rabbit anti-aSMase, anti-nSMase, anti-TNFR1, or anti-TNFα antibodies (ab83354, ab131330, ab19139, and ab6671, Abcam Cambridge, UK) in 5% (*w/v*) bovine serum albumin (BSA) in 1× TBS-Tween 20 (0.1% (*w/v*) Tween 20 in 10 mM Tris, pH 7.5) at +4 °C with shaking. HRP-conjugated goat anti-rabbit antibodies (ab97051, Abcam) were added and incubated for 60 min at room temperature. Membranes were then exposed using the 3,3′-diaminobenzidine (DAB) detection and the obtained images were analyzed by the ImageJ tool kit. 

SMase activity was determined in soleus muscle homogenates. For that, the aSMase assay kit was used according to the described protocol (ab190554, Abcam). Briefly, muscle homogenate samples were added to 50 μL of sphingomyelin working solution in 96-well black plates and incubated at 37 °C for 2 h, after which 50 μL of sphingomyelinase assay solution was added. The mixture was incubated for 1 h at room temperature. Fluorescence was measured by the microplate reader Stat Fax 2100 (Awareness Technology, Palm City, FL, USA) at 545 nm. The fluorescence in the blank wells with the assay buffer was only used as a negative control.

### 4.4. Immunohistochemistry 

Under anesthesia, animals were perfused through the ascending aorta with phosphate-buffered saline (PBS in mM: 3.2 NaH_2_PO_4_, 0.5 K_2_HPO_4_, 1.3 KCl, 135 NaCl, pH 7.4) and then with 4% paraformaldehyde prepared with PBS. All these reagents were from Sigma. Soleus muscles were postfixed in the same fixative for 2 h, transferred to a 30% sucrose solution for a day, and frozen on dry ice. Using a Shandon Cryotom E (Cheshire, UK), serial muscle sections (14 µm) were prepared and mounted on Superfrost Plus slides (Thermo Fisher Scientific, Waltham, MA, USA). Before staining, sections were kept for 10 min in 3% hydrogen peroxide. After washing in PBS, the sections were incubated for 2 h in 5% bovine serum albumin solution. For immunofluorescent staining, muscle sections were incubated with anti-Cer monoclonal antibodies (mouse IgG, 1:300, ALX-804-196-T050, Enzo Life Sciences, Farmingdale, NY, USA) and then with anti-mouse biotinylated antibodies (goat IgG, 1:200, ab64255, Abcam), both for 24 h at room temperature. After washing in PBS, mouse streptavidin-conjugated antibodies (ab191338, Abcam) with goat anti-mouse (FITC) IgG (1:100, ab6785, Abcam) were added and the samples were incubated overnight. The analysis of images made by the Canon PowerShot 600 photo attachment combined with a Nikon Eclipse E200 microscope was performed using the ImagePro Plus 6.0 morphometric program and the ImagePro Insight program (Media Cybernetics, Bethesda, MD, USA). The expression level of immunoreactive ceramide was estimated by the intensity of the fluorescence of the investigated slice on a standard area (0.1 mm^2^). Every fifth section of the organ was taken and 10 intensity measurements were made (at least 150 measurements per animal). Mean values are presented in arbitrary units of gray intensity of binary images used by the mentioned program. 

### 4.5. Fluorescence Microscopy

Fluorescence was acquired using an Olympus BX51WI microscope equipped with a confocal attachment Disk Speed Unit and LumPlanPF100xw objective. The images were captured with a DP71 (Olympus Tokyo, Japan) CCD camera and then were analyzed using ImagePro software (Media Cybernetics, Bethesda, MD, USA). The fluorescence was calculated as the average fluorescence intensity in arbitrary units from pixels in regions of interest. Analysis of junctional fluorescence was conducted in the region defined by α-Btx staining. Extrajunctional fluorescence was calculated for an area (~200 μm^2^) of sarcolemma outside of the α-Btx-positive region [17]. BODIPY-Cer (or AlexaFluor-488)/α-Btx fluorescence was excited by light of 488/555 nm wavelength and emission was recorded using bandpass filters 505–545/610–650 nm. For the Cer stainings, we used 15–30 muscle fibers to estimate means and SEMs, with number of animals (six) as a sample size (*n*).

#### 4.5.1. Staining with BODIPY-Cer

For direct indication of Cer accumulation in the plasma membranes and intracellular membranes, an exogenous green-fluorescent BODIPY^TM^ FL C5-Cer complexed to bovine serum albumin (B22650, ThermoFisher, Waltham, MA, USA) was used [53,54,55]. BODIPY-Cer was diluted in physiological solution (final concentration 0.1 μM). Then, muscles were perfused for 1.5 h with the BODIPY-Cer-containing (1 mL/min) solution to incorporate the fluorescent Cer into the membranes. An additional washing step (at a perfusion rate 5 mL/min) for 1.5 h was applied to remove unbound fluorescent Cer. Junctional and extrajunctional compartments of the muscle were imaged. Additional staining with rhodamine-conjugated α-Btx (100 ng/mL, T1175T, ThermoFisher), a specific marker of postsynaptic nicotinic acetylcholine receptors (nAChRs), helped to identify the junctional region. α-Btx was added to the bath solution for 15 min immediately after exposure to BODIPY-Cer.

#### 4.5.2. Immunolabeling of Plasma Membrane Cer

After fixing in fresh 3.7% paraformaldehyde in PBS (for 20 min), the neuromuscular preparations were blocked with 2% normal goat serum and 3% BSA in PBS at room temperature for 2 h. The samples were subsequently incubated with primary mouse monoclonal anti-Cer antibody (1:50, ALX-804-196-T050, Enzo Life Sciences, Farmingdale, NY, USA) for 2 h at room temperature, without the permeabilization step. Postsynaptic nAChRs were labeled by exposing the fixed tissue to 1 ng/mL of rhodamine-conjugated α-Btx. Then, the muscles were incubated with secondary AlexaFluor-488-labeled anti-mouse antibody (1:2000, ab150113, Abcam) for 1 h at room temperature in the dark. The samples were then placed onto glass slides and sealed with mounting medium for confocal microscopy. Both the primary and secondary antibodies were diluted in PBS containing 2% normal goat serum and 2% BSA. After each step of the labeling protocol, the preparations were washed four times with PBS over 1.5 h. For negative controls, some samples were incubated only with the secondary antibody and no specific staining was detected (Figure S2).

#### 4.5.3. Monitoring Membrane Asymmetry

F2N12S (4’-N,N-diethylamino-6-(N,N,N-dodecyl-methylamino-sulfopropyl)-methyl-3-hydroxy- flavone (A35137T, ThermoFisher)) is a violet excitable fluorescent probe for detection of the loss of the plasma membrane asymmetry occurring during early steps of cell death [70]. This ratiometric probe reacts to changes in the charge on the outer leaflet of the plasma membrane. Muscles were incubated with F2N12S (200 nM) for 5 min and then washed with normal physiological solution for 10 min. After that, the dye fluorescence was captured within 20 min. The dye fluorescence was excited by a 405 ± 15 nm light and emission was detected at 530 ± 30 nm (green fluorescence) and 585 ± 30 nm (orange fluorescence). The ratio of Or/Gr fluorescence was calculated. Loss of membrane asymmetry (a hallmark of membrane disturbance) causes a decrease in the Or/Gr ratio. This is linked with changes in the charge of surface membranes due to translocation of anionic phospholipids (phosphatidylserine and phosphatidyl-ethanolamine), which normally reside in the inner leaflet of the plasma membrane, to the outer leaflet of the plasma membrane.

### 4.6. Statistics

Statistical analysis was performed using Origin Pro software (Northampton, MA, USA) and Statistica 6.0 (StatSoft Inc.,Tulsa, OK, USA). Data in the text are presented as mean ± SEM. Statistical significance of the difference between group means was evaluated using one-way ANOVA followed by the Bonferroni post hoc test or Mann–Whitney U test (for 3.1 and 3.2). *p*-Values < 0.05 were considered significant.

## Supplementary Materials

Supplementary materials can be found at https://www.mdpi.com/1422-0067/20/19/4860/s1.

## Abbreviations

α-Btx	rhodamine-conjugated α-bungarotoxin
aSMase	acid sphingomyelinase
Cer	ceramide
HS	hindlimb suspension
nAChR	nicotinic acetylcholine receptor
nSMase	neutral sphingomyelinase
TNFα	tumor necrosis factor α
TNFR1	TNFα receptor 1
