# Supplementary material for: Changes in Membrane Ceramide Pools in Rat Soleus Muscle in Response to Short-Term Disuse
Source: Int J Mol Sci. 2019 Sep 30;20(19):4860. doi: 10.3390/ijms20194860 (PMC6801848; doi:10.3390/ijms20194860)
Supplement: Supplementary file 1 [file ijms-20-04860-s001.pdf]

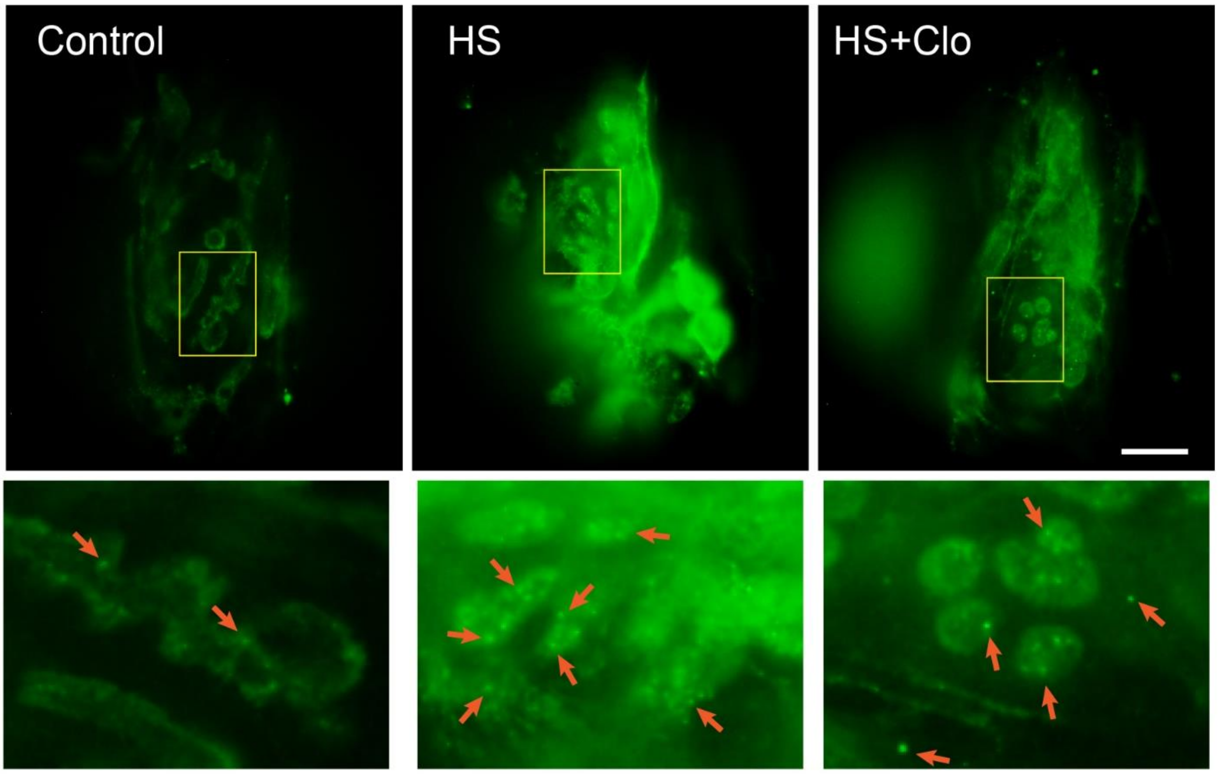

Figure S1. Hindlimb suspension (HS) stimulates appearance of ceramide (Cer) immunoreactive spots and vesicle-like structures in the plasma membranes of junctional regions. The top row, the representative fluorescent images of Cer fluorescence in control, suspended muscle (HS) or suspended muscle of clomipramine treated rats (HS+Clo). Scale bar - 10 µm. Regions selected in rectangles were rotated to 90° and shown in the bottom row. Arrows indicate the fluorescent spots.

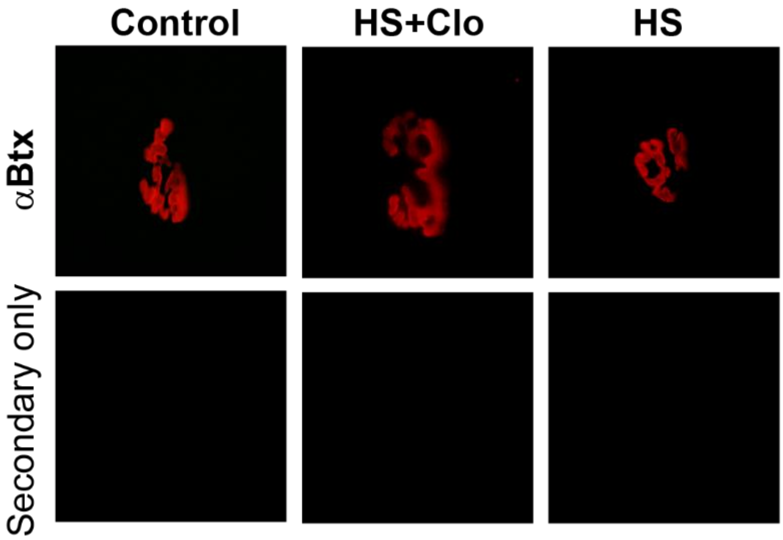

Figure S2. Negative controls for immunofluorescent staining of plasma membrane ceramide. After fixing, the muscles were incubated with blocking solution at room temperature for 2h. The samples were subsequently incubated with PBS containing 2% normal goat serum, 2 % BSA and rhodamine-conjugated α-Btx (1 ng / ml) for 2 h at room temperature. Then the muscles were incubated with secondary AlexaFluor-488 labeled anti-mouse antibody (1:2000) for 1 h at room temperature in the dark. Secondary antibodies were diluted in PBS containing 2% normal goat serum and 2 % BSA. After each step of the labeling protocol the preparations were washed four times with PBS over 1.5 h.
